# Biodistribution and racemization of gut-absorbed l/d-alanine in germ-free mice

**DOI:** 10.1038/s42003-023-05209-y

**Published:** 2023-08-16

**Authors:** Tian (Autumn) Qiu, Cindy J. Lee, Chen Huang, Dong-Kyu Lee, Stanislav S. Rubakhin, Elena V. Romanova, Jonathan V. Sweedler

**Affiliations:** 1 Department of Chemistry, University of Illinois Urbana-Champaign, Urbana, IL USA; 2 Beckman Institute, University of Illinois Urbana-Champaign, Urbana, IL USA; 3 Neuroscience Program, University of Illinois Urbana-Champaign, Urbana, IL USA; 4 Present Address: Department of Chemistry, Michigan State University, East Lansing, MI USA; 5 Present Address: College of Pharmacy, Chung-Ang University, Seoul, Republic of Korea

**Keywords:** Cellular neuroscience, Liquid chromatography

## Abstract

Microbiome-derived metabolites are important for the microbiome-gut-brain axis and the discovery of new disease treatments. d-Alanine (d-Ala) is found in many animals as a potential co-agonist of the *N*-methyl-d-aspartate receptors (NMDAR), receptors widely used in the nervous and endocrine systems. The gut microbiome, diet and putative endogenous synthesis are the potential sources of d-Ala in animals, although there is no direct evidence to show the distribution and racemization of gut-absorbed l-/d-Ala with regards to host-microbe interactions in mammals. In this work, we utilized germ-free mice to control the interference from microbiota and isotopically labeled l-/d-Ala to track their biodistribution and racemization in vivo. Results showed time-dependent biodistribution of gut-absorbed d-Ala, particularly accumulation of gut-absorbed d-Ala in pancreatic tissues, brain, and pituitary. No endogenous synthesis of d-Ala via racemization was observed in germ-free mice. The sources of d-Ala in mice were revealed as microbiota and diet, but not endogenous racemization. This work indicates the importance of further investigating the in vivo biological functions of gut-microbiome derived d-Ala, particularly on NMDAR-related activities, for d-Ala as a potential signaling molecules in the microbiome-gut-brain axis.

## Abstract (web summary)

The microbial metabolite, d-alanine, cannot be endogenously synthesized by mice, and is instead derived from the food and gut microbiota, with gut-absorbed d-alanine accumulating in the mouse pancreas, brain, and pituitary.

## Introduction

Investigation of chemical interactions in the microbiome-gut-brain axis has become an increasingly important for the understanding and treatment of neurological diseases such as autism spectrum disorder^1^, Alzheimer’s disease^2^, and Parkinson’s disease^3^. Microbiome-derived metabolites can be involved in cell-cell chemical signaling in different tissues and organs of the host including the endocrine and central nervous system. The gut microbiome is known as a source of a variety of bioactive molecules such as trimethylamine-*N*-oxide linked to cardiovascular health^4^, phenolic metabolites that reduce neurodegeneration^5^, short-chain fatty acids related to metabolic diseases^6^, and neurotransmitters such as γ-aminobutyric acid that can regulate the enteric nervous system^7^. Discovery and characterization of functional microbiome-derived metabolites are thus important for future microbiome-based disease interventions.

d-Alanine (d-Ala) is a poorly characterized but intriguing potential microbial signaling molecule in the microbiome-gut-brain axis. d-Ala is an essential component of the bacterial cell walls, produced from l-Ala by microbial alanine racemases and exists almost ubiquitously in the realm of microorganisms^8^. Like d-serine (d-Ser), d-Ala interacts with the glycine binding site of the *N*-methyl-d-aspartate receptors (NMDARs)^9,10^. NMDAR is an ionotropic glutamate receptor expressed in different cell types including neurons where it is mainly associated with excitatory synapses^11^. d-Ser modulates NMDAR activity in the central nervous system thus impacts brain functions demonstrating clinical relevance to neurological disorders such as depression and schizophrenia^12–14^. Similarly, d-Ala has been shown to be a potent, stereo-selective co-agonist of NMDAR in vitro^9,10^. Using *Caenorhabditis elegans* as the animal model, researchers showed that d-Ala can regulate animal behavior via the NMDAR^15^. In higher animals, although in vivo evidence of d-Ala function is currently lacking and under investigation, evidence suggests d-Ala may be involved in a range of functions, particularly in the nervous and endocrine systems. As examples, d-Ala levels fluctuate in a circadian manner in both rodents and human tissues^16–19^. As with several other d-amino acids (d-AAs), d-Ala is detected in endocrine structures including the insulin-secreting β-cells in the pancreatic islets and adrenocorticotropic hormone-secreting cells in the pituitary gland^20–23^. In addition, changed d-Ala levels were found in samples from individuals with various diseases such as Alzheimer’s disease, diabetes, renal diseases, cancers, and more, as summarized in our previous review^24^. This makes d-Ala a potential biomarker and pharmacological target for these diseases.

Surprisingly given this interest, the origin of the d-Ala in animals, particularly in widely used rodents, is not known. There are several potential sources of d-Ala in animals—diet, gut microbiota, and endogenous synthesis. Diet, especially fermented and processed food, contains d-Ala and can be a source of d-Ala^25^. The microbial origin of d-Ala is supported by a number of evidence. Our work showed d-Ala production by isolated gut microbial species^26^. Other groups have shown the expression of alanine racemases in microorganisms^27,28^, and dependence of d-Ala levels on the presence of the gut microbiota^18,29^. Although no endogenous alanine racemases have been identified in mammals, an interesting hypothesis focuses on the potential source of d-Ala in saliva and salivary glands^30,31^. These three factors—diet, gut microbiota, and hypothetical endogenous synthesis—are intertwined, making it hard to differentiate the origins of d-Ala in animal tissues and organs.

**Fig. 1:**
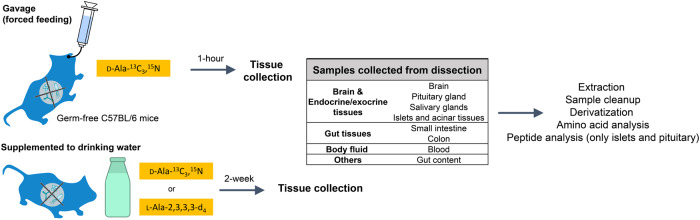
Illustration of experimental design. The experimental scheme for determination of l/d-alanine biodistribution and racemization. Two oral administration scenarios, gavage and drinking water supplementation, are illustrated, followed by a table describing samples collected for amino acid analysis.

In this study, we utilized the germ-free (no microbiota) and conventionally raised mice (with normal microbiota) both orally fed with stable isotopically labeled d/l-Ala. Main goals of the study are to (1) identify the origins of d-Ala in mouse, (2) track gut-absorbed d-Ala biodistribution, and (3) provide information on the biological functions of d-Ala (Fig. 1). The utilization of germ-free mice eliminated microbiota as a source of d-Ala including related bacterial alanine racemase activity. Stable isotope-labeled Ala provided direct evidence of d-Ala gut absorption and biodistribution as well as the absence of endogenous l-Ala racemization in our experimental time frame. In the experiments, solutions containing d- or l-Ala were orally administered via one-time gavage (1-h exposure) or by supplementing drinking water with these isotopes for two weeks. These two experimental scenarios explore the correlation of the metabolite’s biodistribution to short- and long-term exposures of animals to exogenous l/d-Ala. To explore the potential endogenous synthesis of d-Ala via racemization, stable isotope labeled l-Ala was fed to germ-free and conventional mice and the d-Ala isotope product was monitored. Chiral derivatization of amino acids (AAs) using a modified version of Marfey’s reagents was used to separate the d-/l-Ala enantiomers extracted from samples. Concentrations of unlabeled and isotope-labeled Ala were measured using an ultra-performance liquid chromatography coupled to a triple quadrupole mass spectrometer. Lastly, to provide initial insights on the potential biological impact of d-Ala, peptide profiles in islets and pituitary gland were characterized by matrix-assisted laser desorption/ionization (MALDI) time-of-flight (TOF) mass spectrometry. Here we confirmed the sources of d-Ala in mammals and revealed the biodistribution of d-Ala through gut absorption, paving the way for future functional studies of this potential signaling molecule in the microbiome-gut-brain axis.

## Results

### Differential sample-specific levels of endogenous l/d-Ala in mouse food, germ-free and conventional mice

**Fig. 2:**
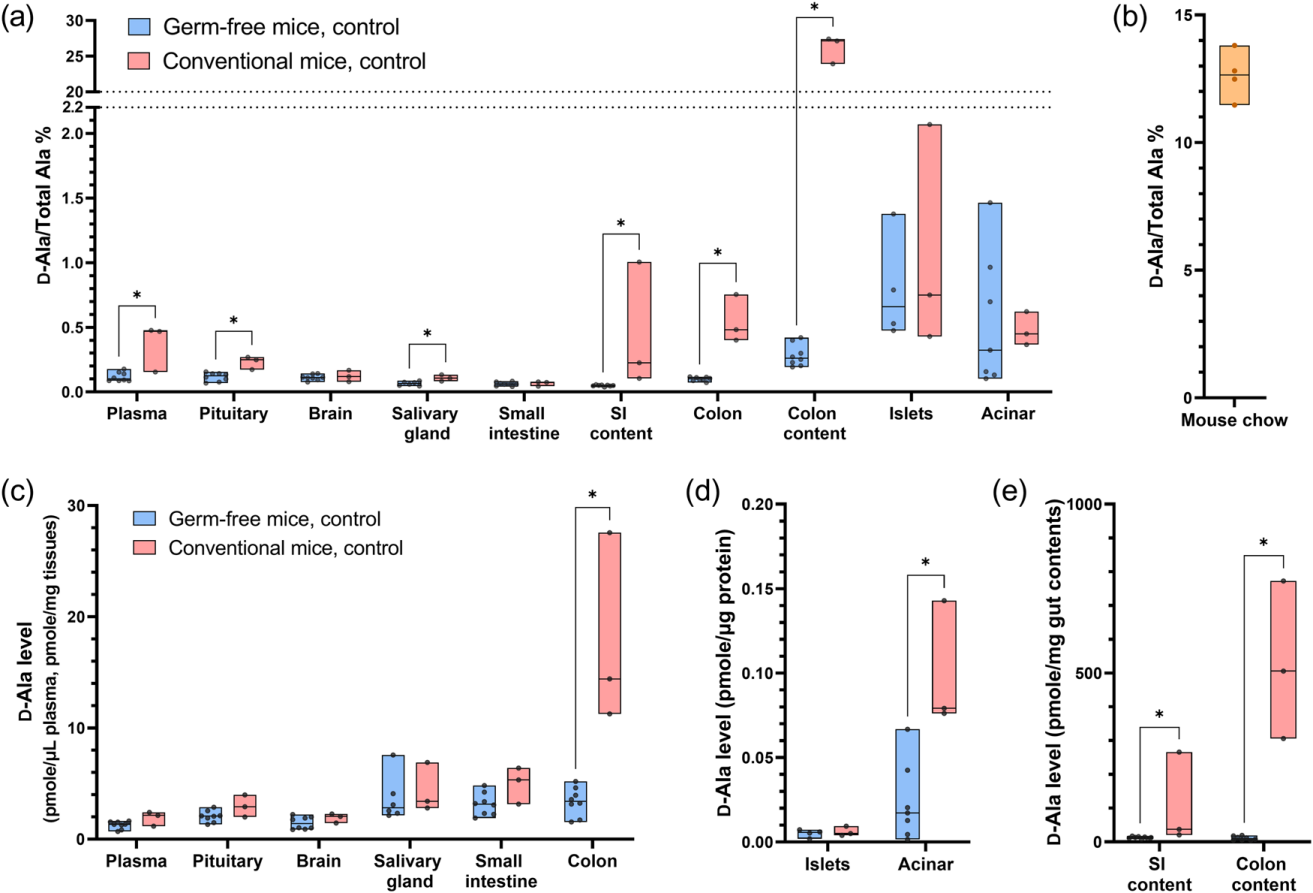
Endogenous, unlabeled d-Ala levels in different tissues. **a** d-Ala percentages (d-Ala/(l-Ala+d-Ala)%) and (**c**–**e**) d-Ala concentrations are shown in samples from germ-free (*n* = 8) and conventional mice (*n* = 3), as well as the percentages in mice chow (**b**) (*n* = 4). Asterisks indicate the pairwise comparisons with statistical significance (α = 0.05) from Mann–Whitney tests. Each data point represents one sample from one individual animal; for mouse chow, four data points represent four samples taken from two batches of food (1 and 3 samples for each batch). Floating bars indicate minimum and maximum, and the middle lines indicate median. SI small intestine.

Measurements of endogenous, unlabeled free d- and l-Ala were performed in the animal samples as well as mouse chow (Fig. 2). d-Ala percentages (d-Ala/(l-Ala+d-Ala)%) for animal samples (Fig. 2a) and mouse chow (Fig. 2b) were calculated, and plotted together with d-Ala concentrations (Fig. 2c–e). To compare d-Ala% and d-Ala levels in each type of sample, pairwise comparisons were individually performed between germ-free and conventional mice. We found that the plasma, pituitary, salivary gland, small intestine content, colon, and colon content showed higher d-Ala% in conventional mice compared to germ-free mice. However, the trends for d-Ala concentration in samples were not always consistent with d-Ala%. Only acinar tissues, colon, and gut contents showed higher d-Ala concentrations. Among all samples, colon contents from conventional mice showed the highest d-Ala%. Pancreatic islets and acinar tissues were among the highest in the measured tissue types in both germ-free and conventional mice. While islets and acinar tissues showed similar d-Ala% in germ-free and conventional mice, acinar tissues showed higher d-Ala concentrations in conventional mice. Interestingly, the d-Ala concentration in conventional mice was significantly higher than that in germ-free mice while d-Ala% was similar, indicating a similar trend of l-Ala in the acinar tissues of conventional mice.

### Biodistribution of gut-absorbed d-Ala-^13^C_3_,^15^N in germ-free mice

Two d-Ala-^13^C_3_,^15^N oral administration scenarios were implemented, a one-time gavage of ~40 mg with 1-h waiting time and a two-week feeding of ~100 mg d-Ala-^13^C_3_,^15^N, estimated by water consumption. d-Ala-^13^C_3_,^15^N levels in various samples were measured and calculated. Blood from tissues was removed via transcardiac perfusion at tissue collection. Concentrations of d-Ala-^13^C_3_,^15^N in plasma/tissue/gut contents were calculated from LC-MS/MS data analysis, as stated in the Method section.

**Fig. 3:**
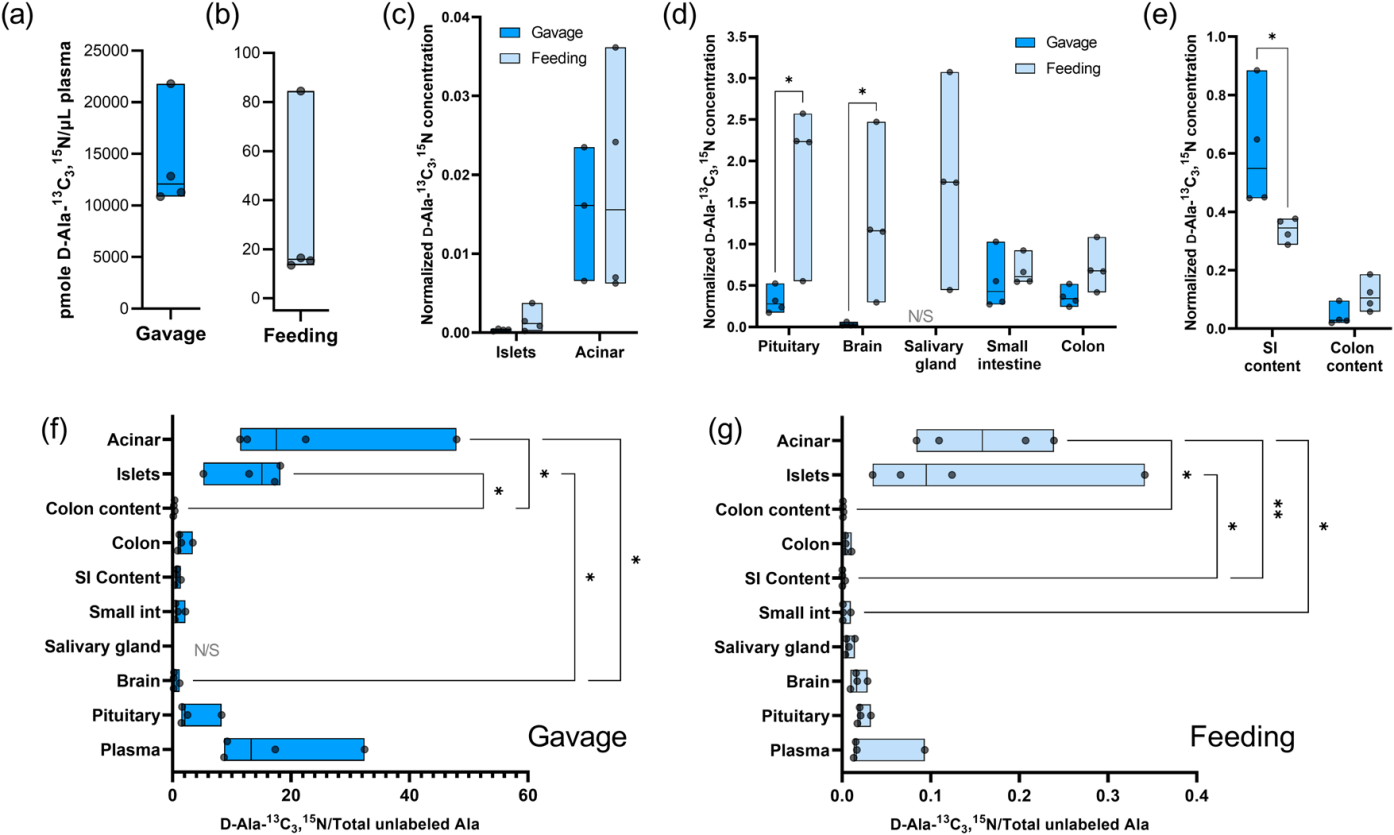
Biodistribution of orally administrated d-Ala-^13^C_3_,^15^N in germ-free mice. **a**, **b** Concentrations (pmole/μL) of d-Ala-^13^C_3_,^15^N detected in plasma of animals orally administrated with d-Ala-^13^C_3_,^15^N via gavage or feeding. **c**–**e** Normalized d-Ala-^13^C_3_,^15^N concentrations (Eq. 1) in various sample types. Asterisks indicate statistical significance from Mann–Whitney tests. **f**, **g** Relative d-Ala-^13^C_3_,^15^N level (Eq. 2) in various sample types in (**f**) gavage and (**g**) feeding experiments. Asterisks with brackets indicate statistically significant difference (*p* < 0.05) between sample types from the post hoc Dunn’s multiple comparison after Friedman’s paired ANOVA (**p < 0.01, *p < 0.05). Each data point represents one sample from one individual animal (*n* = 4). Floating bars indicate minimum and maximum, and the middle lines indicate median. Salivary gland results in gavage experiment are not shown in (**b**, **c**) and not included in statistics due to small sample sizes (*n* = 2). N/S: not shown. SI: small intestine.

We first found that the plasma concentration of d-Ala-^13^C_3_,^15^N was drastically different in two oral administration scenarios (Fig. 3a, b). Thus, to enable comparisons between the two administration models, we normalized the d-Ala-^13^C_3_,^15^N concentration in tissues/gut contents to the d-Ala-^13^C_3_,^15^N concentration in the plasma of the same animal (Eq. 1 in Method). Such normalization strategy was supported by the visual observation and Pearson correlation results that showed mostly positive correlations between plasma d-Ala-^13^C_3_,^15^N level and tissue/gut content d-Ala-^13^C_3_,^15^N level, indicating that the plasma d-Ala-^13^C_3_,^15^N level can be used to represent the dosing scenario and to normalize biological variations (Supplementary Fig. 1, Supplementary Table 1). Pairwise comparisons of normalized d-Ala-^13^C_3_,^15^N concentration between gavage and feeding of the same sample type were performed (Fig. 3c–e). Results showed that normalized d-Ala-^13^C_3_,^15^N concentrations in pituitary and brain were statistically significantly higher in the feeding group than in the gavage group, while interestingly, the small intestine contents showed lower normalized d-Ala-^13^C_3_,^15^N concentrations in the feeding group. We also compared the relative d-Ala-^13^C_3_,^15^N levels across sample types (Fig. 3f, g). Normalizing the d-Ala-^13^C_3_,^15^N concentration to unlabeled total Ala concentration generated a unitless metric for relative d-Ala-^13^C_3_,^15^N level and enabled cross-sample comparisons within the same administration scenario (Eq. 2). A paired Friedman’s test was used to match the tissues from the same animal in the analysis of variance (ANOVA) analysis. ANOVA results showed that sample type had a statistically significant effect on the relative d-Ala-^13^C_3_,^15^N level in both the gavage (*p* = 0.0002) and the feeding groups (*p* < 0.0001). In both administration models, islet and acinar tissues significantly contributed to the observed variances and statistically significantly higher than several other sample types. The relative d-Ala-^13^C_3_,^15^N levels in all sample types in the gavage model are approximately two orders of magnitude higher than those in the feeding model, consistent with the observation with plasma (Fig. 3a, b). We also tested another normalization strategy by further normalizing to the plasma relative d-Ala-^13^C_3_,^15^N level (Eq. 3) and found similar results (Supplementary Fig. 2).

### Effect of d-Ala on peptide levels in pancreatic islets and pituitary

The effect of l- and d-Ala administration on germ-free mice was explored by mapping the biologically active peptide profiles in pituitary and pancreatic islets. The extraction procedure for amino acids also extracted peptides from the same pancreatic islets and pituitary samples. We used MALDI-TOF MS as it only requires a small portion of the limited sample amount available; we were able to detect and semi-quantify multiple known biologically active peptides from the same samples that were used for amino acid analysis. Peptide lists were built by matching the acquired mass lists to the predicted peptide mass from UniProt and results from previous peptidomics studies of the pancreatic islets and pituitaries, including data from our own group^32–38^. Detected peptides (Supplementary Data 1) are assigned to known prohormones that were reported to be expressed in islets or pituitary glands, including insulin 1, chromogranin A, islet amyloid polypeptide, somatostatin, glucagon, provasopressin, and proopiomelanocortin. More importantly, we observed multiple peptides from the same prohormone via the peptide mass fingerprint approach^39,40^, which increased the confidence of both prohormone identification and peptide assignment as demonstrated in numerous studies^38,41,42^.

**Fig. 4:**
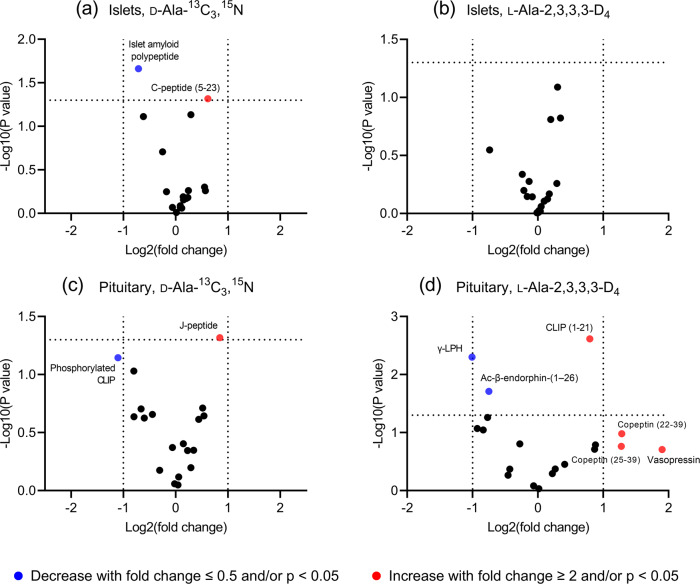
Peptide profile differences after 2-weeks of d-Ala-^13^C_3_,^15^N or l-Ala-2,3,3,3-D_4_ treatment in drinking water compared to control (water only) (*n* = 4). **a**, **b** Pancreatic islets. **c**, **d** Pituitary gland. Volcano plots showed results of uncorrected multiple t-tests between treated and control groups. Each data point represents the fold change and *p* values of log2-transformed, normalized peak intensities in the samples from treated animals compared to control. Data points with larger than 2-fold absolute fold change and/or *p* values smaller than 0.05 are marked with assigned peptide names and color coded (blue for decrease and red for increase in the treated group compared to control). CLIP: corticotropin-like intermediate peptide. J-peptide: joining peptide.

The volcano plots in Fig. 4 show the differences in peptide profiles of the pancreatic islets and pituitary after the 2-week addition of stable isotope labeled l- and d-Ala into drinking water. Borderlines for log2 (fold change) were drawn at -1 and 1 and for *p*-values were placed at -log(0.05). Individual unpaired, two-tailed t-tests were used to compare the normalized intensity of peptide signals in control and treated groups. Potential false discoveries should be kept in mind as no multiplicity was corrected. In general, stable isotope-labeled l- and d-Ala resulted in different responses in peptide levels. After d-Ala-^13^C_3_,^15^N treatment, islets showed statistically significant decreased level of islet amyloid polypeptide and increased level of C-peptide (Fig. 4a). No similar changes were found in l-Ala-treated groups of pancreatic islets (Fig. 4b). In pituitary after d-Ala-^13^C_3_,^15^N treatment, phosphorylated corticotropin-like intermediate peptide level showed more than 2-fold decrease while J-peptide showed a statistically significant increase (Fig. 4c). l-Ala-2,3,3,3-D_4_ treatment resulted in more changes in the peptide levels in pituitary but quite different from d-Ala-^13^C_3_,^15^N treated groups (Fig. 4d).

### Evaluation of alanine racemase activity in bacterial culture, conventional, and germ-free mice

**Fig. 5:**
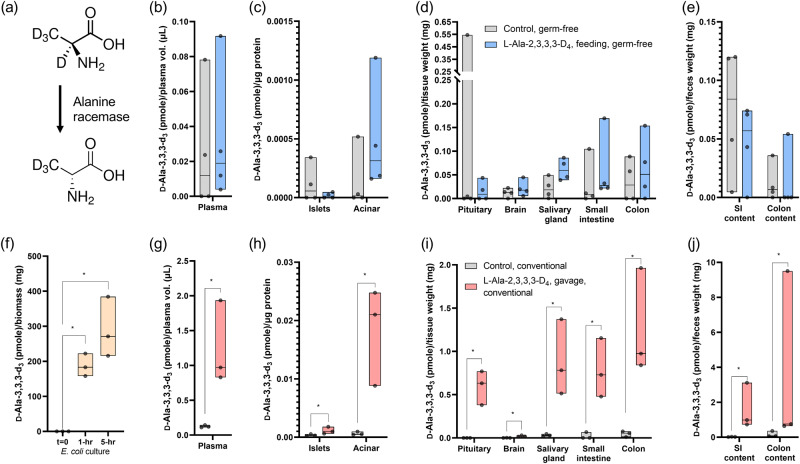
Alanine racemase activities in bacteria, conventional and germ-free mice. **a** Illustration of alanine racemase activity converting l-Ala-2,3,3,3-d_4_ to d-Ala-3,3,3-d_3_. **b**–**e** Concentrations of d-Ala-3,3,3-d_3_ in samples from germ-free mice after two-week feeding of l-Ala-2,3,3,3-d_4_ or regular water as control. **g**–**j** Concentrations of d-Ala-3,3,3-d_3_ in samples from conventional mice 1-h after gavage feeding of solution containing the same stable isotope labeled l-Ala or saline as control. Pairwise comparisons using Mann–Whitney tests (two-tailed for germ-free and one-tailed for conventional mice) were performed between the control and dosed groups individually for each sample type. **f** Racemization of l-Ala-2,3,3,3-d_4_ in bacterial culture. One-tailed Mann–Whitney tests were used to compare between time zero and the two other time points individually. Each data point represents one sample from one individual animal (*n* = 4 for germ-free and *n* = 3 for conventional mice) or one tube of bacterial biomass (*n* = 3). Asterisks with brackets indicate statistical significance (*U* = 0, complete separation). Floating bars indicate minimum and maximum, and the middle lines indicate median. SI: small intestine.

Alanine racemase activity can be detected by monitoring conversion from l-Ala-2,3,3,3-d_4_ to d-Ala-3,3,3-d_3_ (Fig. 5a). d-Ala-3,3,3-d_3_ levels in different samples collected from germ-free mice, conventional mice, and bacterial culture after administration of l-Ala-2,3,3,3-d_4_ were determined from monitoring signals in multiple reaction monitoring (MRM) channels for d-Ala-3,3,3-d_3_ and shown in Fig. 5 after calculations from raw signals to final concentrations. In all animal samples, we observed background signals in the MRM channels for d-Ala-3,3,3-d_3_ surrounding the retention time of d-Ala (Supplementary Fig. 3 as an example), which we believe were from the biological matrices and thus result in the trace levels of d-Ala-3,3,3-d_3_ shown in control samples (gray bars in Fig. 5). Pairwise comparisons between control and treated groups were performed using non-parametric Mann–Whitney tests. No statistically significant differences in d-Ala-3,3,3-d_3_ levels found between germ-free control animals and mice fed with l-Ala-2,3,3,3-d_4_ for 2 weeks, indicating no alanine racemase activities detected in germ-free mice fed with l-Ala-2,3,3,3-d_4_ for two consecutive weeks.

To investigate alanine racemase activity in pure *E. coli* culture, l-Ala-2,3,3,3-d_4_ was supplemented into culture media and d-Ala-3,3,3-d_3_ concentrations in the bacterial biomass were determined. Results showed alanine racemase activities in *E. coli* biomass after 1-h and 5-h incubations leading to time-dependent increase in levels of d-Ala enantiomer (Fig. 5f). Although germ-free mice did not show detectable alanine racemase activities, the transformation of l-Ala-2,3,3,3-d_4_ to d-Ala-3,3,3-d_3_ were readily detected in conventional mice only 1-h after oral gavage of a single high dose of l-Ala-2,3,3,3-d_4_ (Fig. 5g–j). The patterns of relative d-Ala-3,3,3-d_3_ levels (Eq. 2) across sample types were similar to those from gavage experiments of d-Ala-^13^C_3_,^15^N (Supplementary Fig. 4, Fig. 3f), except for in colon and colon contents. Unlike results from gavage experiments with germ-free mice, relative d-Ala-3,3,3-d_3_ levels in colon and colon contents were high, indicating microbial racemase activities in the colon content.

## Discussion

Using germ-free mice and stable isotopically labeled alanine for oral administration, our results have several major conclusions. First, the free d-Ala in mice originates from both food and microbiota, but not endogenous synthesis. Germ-free mice have detectable levels of free d-Ala, indicating that there are sources of d-Ala that are not related to microbiota (Fig. 2a). As the mouse chow has high d-Ala%, food can be a source of free d-Ala (Fig. 2b). Many studies have shown that both raw and processed food contain various levels of free d-AAs, including d-Ala^43^. Comparison between germ-free and conventional mice clearly shows that the existence of microbiota drastically increases the d-Ala levels in colon contents and colon tissue, indicating microbiota-derived d-Ala may be absorbed through the colon (Fig. 2a, c, e). The higher level of d-Ala in colon content compared to small intestine content may be due to a higher density of microbiome in the colon content^44^. d-Ala% in the plasma, pituitary, salivary glands, and small intestine contents are higher in conventional mice compared to those in germ-free mice, although the differences are less obvious or disappeared when looking at d-Ala concentrations (Fig. 2a, c). For plasma, pituitary, and pancreas, we did not see drastic differences in germ-free compared to specific-pathogen free mice as reported by Karawaka, et al.^18^. This could be due to reasons including different animal strains, food of choice, d-Ala analysis measurement techniques, sample type (e.g., entire pancreas vs. isolated acinar tissues), extent of blood removal during sample preparation, etc. For example, Bruckner et al. determined the percentage of d-Ala in rat chow as high as 6.9%^45^, and we measured 12.6% in the mice chow used in our study. Interestingly, the d-Ala levels we measured in this study seem to be among the lowest reported except for in colon contents (Supplementary Table 2).

Our results showed that there was no detectable alanine racemase activity in germ-free mice. Alanine racemases, which convert l-Ala to its d-form, have been found in many microbes and several aquatic invertebrates, but no alanine racemase has been characterized in vertebrates^8,24^. Multisequence alignment of the mouse serine racemase and several selective amino acid racemases from prokaryotes, yeast and invertebrates showed more similarities of mouse serine racemase to broad specificity amino-acid racemases compared to alanine racemases (Supplementary Fig. 5). So far, evidence of endogenous alanine racemase activity in vertebrates is related to saliva and salivary glands. More than 30% of total Ala has been reported to be in d-form in human saliva^30,46^. However, the same studies showed that the d-Ala% were lower in oral tissue cells and in salivary glands, while microorganisms in the oral cavity showed a higher percentage of d-Ala. Low levels of d-Ala were also observed in the rat salivary glands (0.2-0.3%)^31^. These suggest that d-Ala originating from the oral microbiome likely contributes to the d-Ala detected in saliva. Nevertheless, the hypothesis of d-Ala endogenous synthesis cannot be ruled out. Our results clearly showed that after a 2-week oral administration of l-Ala-2,3,3,3-d_4_, no l- to d-Ala transformation was observed (Fig. 5b–e). To exclude the effect of enantiomeric impurities in the standards, the level of d-Ala-3,3,3-d_3_ in the l-Ala-2,3,3,3-d_4_ solution used for oral administration was determined as negligible (~0.1% after autoclave) (Supplementary Table 3). We conclude that no endogenous alanine racemase activities are present in germ-free mice within the timeframe and the treatment dose we evaluated. As microbes can rapidly transform l-Ala-2,3,3,3-d_4_ to its d-form (Fig. 5f), it was not surprising that a short gavage experiment of l-Ala-2,3,3,3-d_4_ performed with conventional mice possessing normal microbiota resulted in detection of alanine racemase activity in all sample types examined (Fig. 5g–j). In fact, the comparison of normalized d-isotope ratio to unlabeled Ala across sample types showed very similar patterns to those observed in the gavage experiment involving d-Ala-^13^C_3_,^15^N (Supplementary Fig. 4; Fig. 3f), except for the colon and colon contents, where d-Ala was produced by microbial alanine racemase activities. Our results strongly support the conclusions that the d-Ala in mouse originated from the microbiota and food, and not by endogenous biosynthesis through enzymatic racemization.

We demonstrated that d-Ala can be absorbed by the intestines and showed the biodistribution of gut-absorbed d-Ala using d-Ala-^13^C_3_,^15^N oral administration in germ-free mice without interference from microbiota. Oral administration of d-Ala in rodents has been done^47–50^, showing increased levels of d-Ala in serum, urine, and some parts of the brain, but not pituitary. However, the alanine racemase activity from the gut microbiota may have hampered an accurate evaluation of the gut-absorbed d-Ala biodistribution. With germ-free mice and d-Ala-^13^C_3_,^15^N, we differentiated the exogenous d-Ala through oral administration and the unlabeled d-Ala from food and excluded the contribution from microbial activities. The two feeding scenarios resulted in a ~400-fold difference in the d-Ala-^13^C_3_,^15^N level in plasma, despite the total consumption of d-Ala-^13^C_3_,^15^N being less in the short-term experiment (~40 vs ~100 mg) (Fig. 3a, b). Assuming similar d-Ala absorption efficiency through gut, this indicated that most of the orally administrated d-Ala-^13^C_3_,^15^N were likely removed from the animal over time. We also determined that blood transported the gut-absorbed d-Ala-^13^C_3_,^15^N to tissues. In both feeding scenarios, the ratio of d-Ala-^13^C_3_,^15^N over total unlabeled alanine in plasma was always among the highest in all samples, except for islets and acinar tissues (Supplementary Fig. 1), and mostly positively correlated to the ratio in other samples (Supplementary Table 1).

We obtained clear evidence of gut-absorbed d-Ala entering the central nervous system and accumulating over time. Normalization of d-Ala-^13^C_3_,^15^N concentration (Eqs. 1–3) enabled comparisons between feeding scenarios and across sample types. Significantly higher levels of d-Ala-^13^C_3_,^15^N are found in the pituitaries (6-fold) and brains (42-fold) of the feeding group exposed to the isotope for 2-weeks compared to the exposed for 1-h gavage group (Fig. 3d). These findings suggest that d-Ala-^13^C_3_,^15^N accumulated in the pituitary and brain over time. The accumulation of d-Ala-^13^C_3_,^15^N in the brain also demonstrates that d-Ala can cross the blood-brain barrier. As d-Ala is a co-agonist of the NMDAR and found to have different concentrations in different parts of the brain, gut-absorbed d-Ala accumulation in the brain over time suggests a mechanism to regulate d-Ala levels, which may be related to its functions. For example, the correlation of d-Ala levels in rodents and humans with circadian rhythm has been reported^16–19^, while the d-Ala concentration is reported to be high in the pineal gland^49^. As both d-Ala and d-Ser interact with the same NMDA receptor binding site, it will be interesting to determine how these two systems interact given one is endogenous and the other an exogenous molecule.

Previous evidence, including immunostaining against d-Ala^20,21^ and detection of d-Ala change upon glucose stimulation^22^, has pointed out the possibility of d-Ala being involved in the regulation of glucose metabolism and pituitary functions. Interestingly, the circadian changes of d-Ala levels seem to also correlate to the feeding parameters and insulin levels^16^ and differential gut absorption of d-Ala^18^. We found that in both short- and long-term oral administration of d-Ala-^13^C_3_,^15^N, the normalized d-Ala-^13^C_3_,^15^N ratio in both the pancreatic islets and acinar tissues were always similar and among the highest measured (Fig. 3f, g), despite that the normalized d-Ala-^13^C_3_,^15^N concentration in the pancreatic islets were much lower than that in acinar tissues (Fig. 3c). The islets and acinar tissues in untreated animals also showed high levels of d-Ala% in both germ-free and conventional mice (Fig. 2a). Together, these findings indicate that the islets and acinar tissues can rapidly uptake gut-absorbed d-Ala and maintain a relatively stable level of d-Ala over time.

Given the high levels of d-Ala in endocrine structures such as islets and pituitary, we ask whether administration of d-Ala impacts peptide hormones. Prior research supports the impact of d-amino acid and peptide dynamics in the pituitary and pancreatic islets. For example, d-aspartate was found to induce the release of prolactin, growth hormone, and luteinizing hormone from the rat anterior pituitary^51,52^. Thus, we performed preliminary measurements on the effects of d-Ala on the pituitary and pancreatic islets collected from the same animals / samples administrated d-Ala-^13^C_3_,^15^N or l-Ala-2,3,3,3-d_4_ for 2 weeks (Fig. 4). Uncorrected unpaired, multiple t-tests were used to maximize the number of molecular features differentiating the peptide profiles. Results showed that the two-week treatment of d-Ala or l-Ala isotopes resulted in differences in the peptide profiles of both pancreatic islets and pituitary glands. Several peptides were found to change levels only in d-Ala-treated groups but not l-Ala-treated groups. In most cases, only one peptide displayed noticeable change of their levels in treated groups, while other peptides from the same prohormone remained the same. For example, out of the detected peptides from the proopiomelanocortin prohormone, only phosphorylated corticotropin-like intermediate peptide known to function as an insulin secretagogue in the pancreas^53^ and joining peptide with unknown biological roles showed noticeable changes in their levels. More interestingly, the directions of change were opposite for these two peptides (Fig. 4c). The biological importance of these observations requires further investigation with a more quantitative method for a more comprehensive understanding of d-Ala influence on peptide dynamics.

In terms of its function, both d-Ser and d-Ala are effective co-agonists of the NMDA receptor. While d-Ser is a known endogenous neuromodulator, here we demonstrated that d-Ala is not locally synthesized but derives from the gut microbiome and diet by measuring the accumulation and conversion of stable isotopically labeled d-/l-Ala after oral administration in germ-free and conventional mice. We found that pancreatic islet and acinar tissues had the highest levels of gut-absorbed d-Ala isotopes, and pituitary and brain tissues accumulated gut-absorbed d-Ala over time. Profiling the peptide level change in pituitary and islets pointed out the potential involvement of gut-absorbed l- and d-Ala in the functions of nervous and endocrine systems. We conclude that microbiota and diet are two sources of d-Ala in mice, while no endogenous synthesis of d-Ala via endogenous racemization was observed over the time period of our experimental design. Our results imply that NMDA receptor activity depends on a balance of endogenously synthesized d-Ser and exogenous diet/gut microbiome-derived d-Ala, which may allow a unique metabolic interplay among the microbiota, diet, and the nervous and endocrine systems to control NMDA receptor activity.

Meanwhile, several limitations in this study outline questions to be addressed by further studies. The usage of germ-free mouse poses challenges in understanding the role of d-Ala under a normal physiological condition as the germ-free mice show biochemical and physiological abnormalities compared to conventionally raised animals. Better quantification of neuropeptides and hormones are needed for a more comprehensive understanding of the impact of d-Ala on peptide dynamics, and more details on d-Ala transporters are needed to understand how d-Ala is taken up by various nervous and endocrine systems. The limited number of animals and only two time points were used due to the expense of the stable isotopic labels and germ-free animals; however, a time-course study will be able to provide more insights into the pharmacokinetics of gut-absorbed d-Ala. Finally, with the discovery of d-Ala accumulation in the brain, further studies are needed to investigate the distribution of gut-absorbed d-Ala in different brain regions.

## Methods

### Materials and reagents

d-Ala-^13^C_3_,^15^N (Cat. #: 760277) and d-Ala-3,3,3-d_3_ (Cat #: 642975) isotopes were obtained from Sigma Aldrich. l-Ala-3,3,3-d_3_ (Cat #: DLM-248-1), and l-Ala-2,3,3,3-d_4_ (Cat. #: DLM-250-PK), l-Leu-5,5,5-d_3_ (Cat. #: DLM-1259), and l-Ser-^13^C,^15^N (Cat. #: CNLM-7814) isotopes were obtained from Cambridge Isotope Laboratories. Unlabeled l-Ala was purchased from Fluka and d-Ala was from Sigma Aldrich. A set of 20 G 1.5” reusable stainless feeding needle (N-PK) was purchased from Braintree Scientific, Inc. Methanol (Cat. #: A456) and H_2_O (Cat. #: W64), Optima™ LC/MS Grade, were purchased from Fisher Scientific.

### Animals

Animal experiments were performed following the animal use protocol #19251 approved by the Institutional Animal Care and Use Committee (IACUC) at the University of Illinois Urbana-Champaign (UIUC). Germ-free C57BL/6 male mice (9-11 weeks old) were obtained from the Rodent Gnotobiotic Facility at the UIUC. Conventional C57BL/6 inbred male mice (8 weeks old) were purchased from Envigo and housed in the animal facility at the Beckman Institute at UIUC. All mice were housed as 2 animals per cage. Both germ-free and conventional mice were fed with sterilized 2019S Teklad Global 19% Protein Extruded Rodent Diet (Sterilizable) from Envigo. As males and females need to be separately investigated due to their different hormone profiles and cycles, we decided to perform this study only using male mice.

### Oral administration of stable isotopes

Due to the high price of germ-free mice and stable isotopes we used, we chose to use the minimal acceptable sample size for statistics (*n* = 3 or 4 for each group) and investigate whether effects can be seen with a small sample size. Cages of mice were randomly chosen to perform control or oral administration of stable isotopes. Every experiment of oral administration consisted of an equal number of control and treated animals (*n* = 2 for germ-free mice, *n* = 3 for conventional mice). The experiment of oral administration to germ-free mice was performed twice on different days and the results were combined to have a total sample size of 4.

#### Gavage

Solutions of 0.9% NaCl and 150 mg/mL d-Ala-^13^C_3_,^15^N were made and filtered with 0.22 μm PVDF sterile filter. With proper sterile procedures, sealed positive pressure individual ventilated cages holding 10-week-old germ-free C57BL/6 mice, as well as solutions and tools, were transferred to a biosafety cabinet. All materials were autoclaved/sterile filtered previously and sanitized immediately before sending into the BSC through a specifically designed portal. Each germ-free mouse was weighed inside of the biosafety cabinet and orally administrated with 260–270 μL (10 mL/kg body mass) of saline or d-Ala-^13^C_3_,^15^N solution (1.5 g/kg body mass) in the biosafety cabinet, transferred back to its original cage, and sent back to the rack. To minimize the impact of circadian rhythm on alanine levels in mice, gavage experiments were performed around the same time each day (~11 am). After 60 min, the mice were sent for CO_2_ euthanasia and dissection. Each gavage experiment included 4 mice (2 for saline and 2 for d-Ala-^13^C_3_,^15^N). The experiment was repeated twice. For conventional animals treated with l-Ala-2,3,3,3-d_4_, animals were fed with the same food as germ-free mice for 2 weeks after they arrive at our facility before experiments, and solutions of 0.9% NaCl and 150 mg/mL of l-Ala-2,3,3,3-d_4_ were orally administrated with 10 mL/kg body mass of saline or l-Ala-2,3,3,3-d_4_ (1.5 g/kg body mass). The gavage experiment included 6 mice (3 for saline and 3 for l-Ala-2,3,3,3-d_4_) following the same timeline as the germ-free experiments.

#### Feeding

Solutions of 1.25 g/L d-Ala-^13^C_3_,^15^N and l-Ala-2,3,3,3-d_4_ were made using the same tap water used to feed germ-free mice, autoclaved, sent into the BSC with proper sterile procedures, and used to fill water bottles installed in mice cages. Inside of the BSC, water bottles containing sterile tap water, tap water supplemented with d-Ala-^13^C_3_,^15^N, or tap water supplemented with l-Ala-2,3,3,3-d_4_ were installed into cages already containing 9–10 weeks old mice and the cages were returned to the rack. During the two-week treatment, water levels in the mice cage were monitored by eyes every other day and refilled when necessary. Each feeding experiment included 6 mice (2 for each condition) and the experiment was repeated twice.

### Dissection and sample collection

After treatment time ended, animals were transported to a clean procedure room or a necropsy suite around noon, euthanized by CO_2_ asphyxiation, and followed by tissue collection occurring during afternoon hours. Blood was drawn using a disposable 1 mL syringe directly from the heart after being cut open, transferred into a BD Microtainer tube (Cat. # 365965) and settled on ice. Plasma was separated by centrifugation of the BD Microtainer tube (lithium heparin) at 3000*g* for 10 min at room temperature. Animals were then perfused with cold modified Gey’s balanced salt solution (mGBSS, 1.5 mM CaCl_2_, 4.9 mM KCl, 0.2 mM KH_2_PO_4_, 11 mM MgCl_2_, 0.3 mM MgSO_4_,138 mM NaCl, 27.7 mM NaHCO_3_, 0.8 mM Na_2_HPO_4_, 25 mM HEPES, pH 7.2) through the heart. Mouse pancreata were perfused with a small volume of cold digestive solution (see below for details) and collected for the isolation of islets and acinar tissues. Gastrointestinal tracts, including the small intestine and colon, were cut open longitudinally to collect small intestine and colon contents, and the GI tract tissues were rinsed several times in ice-cold PBS buffer before collection. Brain, salivary glands, and pituitary glands were collected as well. All tissues and gut contents, as well as separated plasma, were set on dry ice immediately after collection and stored at -80C until the next steps.

### Isolation of islet of Langerhans from mouse pancreas

1.06 Wünsch unit of Liberase TL (Sigma) and 0.1 mg/mL of DNase I recombinant (deoxyribonuclease) prepared in Hank’s balanced buffer solution (HBSS, GIBCO) with 5 mM calcium chloride and 25 mM HEPES (Sigma) and sit on ice until use. 2 mL of this enzymatic solution was perfused into the mouse pancreas through the common bile duct. The perfused pancreata were removed from the body and placed in conical tubes with 5 mL of cold Liberase solution on the ice. The tubes were placed in the pre-warmed water bath at 37 °C for 10 min for digestion. At the end of incubation, cold HBSS with 0.2% bovine serum albumin were added into tubes and the tubes were shaken vigorously to ensure the islets were completely separated from the tissue. After the centrifugation at 300*g* for 3 min, the supernatant was removed. These steps were repeated 3 times. At the last step, the supernatant was poured out by decanting and leftover buffer inside the tube were mechanically removed. Subsequently, pellets of digested tissue were resuspended in 7 mL sterile Histopaque 1077 solution (Sigma) with gentle shaking. 3 mL of cold HBSS buffer was layered on the top of the tissue suspension and the tubes were centrifuged at 900*g* for 15 min. The islets located in the supernatants were filtered through 70 μm sterile cell strainer and washed 3 times using Cold HBSS to remove cell debris. The purified islets were placed into a Petri dish filled with cold HBSS, quickly manually picked and transferred to the cold acidified MeOH for analyte extraction (MeOH:H_2_O:acetic acid 90:9:1 by volume).

### Bacteria exposure to isotope and biomass collection

*Escherichia coli* (*E. coli*) strain OP50 were grown on Luria-Bertani agar plates, and single colonies were picked and inoculated into a bottle of 100 mL liquid Luria-Bertani broth for overnight growth. On the next day, aliquots of 5 mL of overnight *E. coli* liquid culture were transferred to sterile culture tubes, and 1 mL of 1.25 g/L sterile l-Ala-2,3,3,3-d_4_ solution or autoclaved water were added. Aliquots of 1 mL bacterial liquid culture were collected before adding isotope and 1- and 5-h after adding isotope. Biomass was collected by centrifugation at 16,000*g* for 1 min, supernatant removed with a 1-mL pipet tip, centrifuged again, the remaining supernatant removed with a 200-μL pipet tip, centrifuged, and formed supernatant removed using a 10-μL pipet tip. Resulting pelleted biomass was weighed and frozen at −80 °C until amino acid extraction.

### Sample processing and analyte extraction

#### Brain/salivary gland/small intestine/colon/mouse chow samples

Frozen samples of the brain, salivary gland, small intestine, colon, or mouse chow were crushed using either a metal tissue pulverizer or a metal hammer on a flat metal surface, and all metal parts were kept cold on dry ice throughout. Crushed cold flakes of samples were then quickly transferred to a 2 mL tissue homogenizing CKMix tube. After weighing, 600 μL of ice-cold 1:1 MeOH:H_2_O was added for tissue homogenization using a Precellys® Evolution tissue homogenizer with a cold trap (Bertin Corp.). Samples were homogenized at 2 × 30s with one 30s pause at 7200 rpm twice, and the resulting suspensions were transferred and centrifuged at 12,000*g* for 20 min at 4 °C. The supernatant was transferred to another tube and dried using a Genevac™ miVac Centrifugal Concentrator. The remaining pellet was added with 600 μL of ice-cold water, followed by vortex, sonication, and centrifugation at 12,000*g* for 20 min at 4 °C. The aqueous supernatant was collected, added to the dried tube previously used for methanol supernatant collection, and dried using the vacuum concentrator. Resulting samples were stored in −20 °C until the next steps.

#### Small intestine content and colon content samples

Frozen samples were thawed, and disposable spatulas were used to scoop and transfer part of sample to a tissue homogenizing CKMix tube. The same sample processing and analyte extraction steps as for brain/salivary gland/intestine samples were followed.

#### Plasma samples

Frozen plasma samples were thawed and 40 μL of plasma was transferred to a new tube. 600 μL of 1:1 MeOH:H_2_O and 600 μL of H_2_O were sequentially added to extract from plasma samples. The resulting supernatants were combined, dried, and stored using the same procedure above.

#### Pituitary samples

A procedure including boiling water, acidified MeOH and 0.25% acetic acid solution was used to extract amino acids and peptides from pituitary gland samples. To the frozen pituitary samples, 400 μL of pre-heated 90 °C water was added, followed by a 10-min incubation in a boiling water bath. Samples were immediately cooled down on ice. A hand homogenizer with disposable plastic pestle was used to disrupt and homogenize samples, followed by centrifugation at 12,000*g* for 15 min at 4 °C. Supernatants were collected, transferred to Low Protein Binding Collection Tubes (Thermo Fisher), and evaporated to dryness using the Genevac concentrator. The same extraction procedure was repeated with 400 μL of acidified MeOH and 400 μL of 0.25% acetic acid, except bath sonication was used instead of hand homogenizer. Dried samples were stored in −20 °C until further use.

#### Islets/acinar tissue samples

Islets and acinar tissues stored in 400 μL of acidified MeOH were homogenized with a hand homogenizer based on disposable plastic pestle, followed by centrifugation at 15,000*g* for 15 min at 4 °C. Supernatants were transferred to a new low protein binding tube. The remaining pellets were extracted by 400 μL of MeOH and 400 μL of water using bath sonication. All three supernatants were combined, mixed, aliquoted, and evaporated to dryness. Dried samples were stored at −20 °C until further use.

#### Cleanup of sample extracts

Extracts of brain, salivary glands, intestines, gut contents, plasma, and mouse chow samples were further processed using centrifugal filters. l-Leu-5,5,5-d_3_ was used as an internal standard to count for the recovery yield at centrifugal filtration. Dried extracts were reconstituted in 300 μL of 33-100 μM l-Leu-5,5,5-d_3_ solution in water, followed by vortexing, sonication, and centrifugation at 16,000g for 15 min at 4 °C. Supernatants were then filtered with a 0.45 µm Nanosep MF centrifugal devices with Bio-Inert® membrane at 10,000*g* for 15 min at 4 °C followed by filtration by a 3 K Nanosep® centrifugal devices with Omega™ membrane at 10,000*g* for 65–75 min at 4 °C. Filtrates were then dried using an Eppendorf Vacufuge concentrator and stored at −20 °C until the next steps.

#### Extraction of analytes from bacterial biomass

Frozen bacterial biomass was thawed and 300 μL MeOH was added to each sample. Biomass was dispersed in MeOH by repetitive pipetting, followed by bath sonication for 10 min or until visibly fully dispersed. The resulting suspension was centrifuged at 16,000*g* for 2 min and the supernatant was collected. The remaining pellets were further extracted with 300 μL water, and the supernatant was collected. Supernatants from two rounds of analyte extraction for the same sample were combined and dried using an Eppendorf Vacufuge concentrator and stored at −20 °C until the next steps.

### MALDI-TOF mass spectrometry peptide profiling and data analysis

Pituitary, islets, and acinar tissue samples were reconstituted in 40 μL of 0.1% trifluoroacetic acid solution in water. Reconstituted samples were centrifuged at 12–14,000*g* for 15 min at 4 °C. 0.5 μL of supernatants was mixed with equal volumes of alpha-cyano-4-hydroxycinnamic acid solution on a sample plate and dried. Another aliquot was taken for BCA assay, and the rest of the extracts were dried and stored at −20 °C. MALDI-TOF MS analysis was performed using an ultrafleXtreme II system (Bruker Daltonics, Billerica, MA) operating in positive mode. Range of *m/z* 800–6000 was examined in reflector mode. Mass spectra were analyzed using the flexAnalysis software (Bruker). Peaks were automatically picked with the Snap peak detection algorithm and an S/N ratio threshold of 6 or manually picked when an observable peak matches a predicted or reported peptide. When only background noise was observed, the peak was not picked, and the data point was marked as at the background level and thus not used for statistics. Identified peptide lists were built by matching the acquired mass lists to the predicted peptide mass from UniProt and results from previous peptidomics studies of the pancreatic islets and pituitaries, including data from our own group^32–38^. A list of identified peptides can be found in Supplementary Data 1. Peak intensities from salt adducts ([M+Na]^+^, [M + K]^+^) were summed up with the intensity of protonated molecular ion [M + H]^+^ to represent the total intensity of the peptide in each spectrum. The summed intensity was then normalized to the total ion current of the spectrum. Uncorrected multiple *t* tests were used to determine the differences in total ion current-normalized peak intensities between the control and treated groups and results were presented in the form of volcano plots to show the fold change values and statistical significance.

### BCA total protein content assay

BCA total protein content assay was performed on the pituitary, islets, and acinar tissue samples using either Pierce™ BCA Protein Assay (Thermo Fisher, Cat. #: 23225) or Micro BCA™ Protein Assay (Thermo Fisher, Cat. #: 23235). A sample volume of 1–2 μL was taken from reconstituted samples prepared for MALDI-TOF MS analysis and diluted according to the manufacturer’s protocol. Calibration curves of 0-40 μg/mL were built for assays.

### Chiral amino acid analysis

#### Marfey’s reaction

A variation of Marfey’s reagent, N_α_-(2,4-dinitro-5-fluorophenyl)-l-valinamide (FDVA, Cat. # 42102, Sigma), was used to react with free amines of analytes present in samples to aid the separation of l/d-Ala using reverse-phase liquid chromatography and analyte quantification using mass spectrometry^54^. A reaction scheme and an example of l/d-Ala separation are shown in Supplementary Fig. 6. The derivatization strategy was chosen due to its higher sensitivity compared to chiral column separation on underivatized alanine. Dried samples were reconstituted in 100 or 120 μL 0.5 M NaHCO_3_ solution (50 μL for pituitary samples, 22 μL for islets/acinar samples). Standards series of l/d-Ala (4-800 μM for l and 0.1-40 μM for d), d-Ala-^13^C_3_,^15^N (10 μM-4 mM), l/d-Ala-3,3,3-d_3_ (2-400 μM for l and 1–200 μM for d), and l-Ala-2,3,3,3-d_4_ (1-400 μM), and l-serine-^13^C,^15^N (4 mM, as internal standard at LC injection) were made in 0.5 M NaHCO_3_ as well. FDVA was weighed using an analytical balance and dissolved in acetonitrile to a final concentration of 5-6 mg/mL (1 mg/mL for islets/acinar samples). Reconstituted samples were centrifuged at 16,000*g* for 5 min at 4 °C, and 10 μL aliquots of supernatants or supernatants diluted into 0.5 M NaHCO_3_ were mixed with 10 μL of FDVA solution in 0.2 mL PCR tubes (except for 20 μL samples and 20 μL FDVA solution for islets and acinar tissue samples). Standards were directly mixed 1:1 with FDVA solution at the same volumes. The mixtures were reacted at 60 °C for 3 h in a Bio-Rad T100 thermal cycler. After the reaction, mixtures were either immediately subject to analysis or frozen at −80 °C until analysis.

#### Desalting and concentrating Marfey’s reaction for islets/acinar tissue samples

Due to the low levels of unlabeled l- and d-Ala extracted from islets and acinar tissue samples, Pierce™ Peptide Desalting Spin Columns (Thermo Fisher, Cat. #: 89851) were used to desalt and concentrate derivatized amino acids from reacted islets and acinar tissue samples. Finished Marfey’s reactions were dried using the Eppendorf Vacufuge concentrator and reconstituted in 150 μL of 0.1% TFA in water, followed by steps in the manufacturer’s protocol with several modifications to maximize the recovery, including a total of 3 times for sample loading and 4 times for sample elution (2x with 50:50 ACN:Water, 0.1% TFA, 2x with 80:20 ACN:Water, 0.1% TFA). Eluted solutions were then dried and stored at −20 °C until analysis.

#### LC-MS/MS analysis

An LC-MS/MS analysis was performed in multiple reaction monitoring (MRM) mode. MRM allows quantitation of the l/d-amino acid contents of the samples. A Bruker Elute ultra-high performance liquid chromatography coupled to a Bruker EVOQ triple quadrupole mass spectrometer operating in negative ESI mode was used in MRM measurements^54^. The MRM channels were built using amino acid standards reacted with FDVA; details of source parameters and MRM transitions can be found in Supplementary Table 4 and 5. Derivatized l/d-amino acids were separated using a Kinetex® 2.6 µm Phenyl-Hexyl 100 Å, 100 × 2.1 mm column (Phenomenex, Cat. #: 00D-4495-AN) at 30 °C. A gradient of 25 mM ammonium formate in water (A) and methanol (B) was used at a flow rate of 0.3 mL/min (0.4 mL/min during 9.5–11 min). The gradient is 0–2 min, 95% A; 2–3.5 min, 95–78% A; 3.5–6 min, 78–50% A; 6–9 min, 50–45% A; 9–9.5 min, 45–10% A; 9.5–11 min, 10% A; 11–11.5 min, 10–95%A; 11.5–12 min, 95% A.

To perform LC-MS/MS analysis, the reaction mixtures were centrifuged at 16,000*g* for 5 min at 4 °C and then diluted 20 to 80-folds (mostly 40-folds) into the loading buffer consisting of 95% mobile phase A, 5% acetonitrile and 2 μM of FDVA-l-Ser-^13^C,^15^N as an internal standard at instrumental injection. Standards were diluted at least 40-folds by the loading buffer. At least two technical sample injections were done for all samples within each LC-MS run. For long continuous LC-MS runs, calibration curves were run at the beginning, in the middle, and at the end of the whole sample list, and a master calibration curve was summarized by averaging all calibration curves. FDVA peak areas were monitored for all samples to calculate the leftover amount of FDVA remaining after Marfey’s reactions and compared to reaction blanks. If the FDVA content in the samples (leftover FDVA) was less than 20% of the reaction blank, indicating potential incomplete reactions, the reconstituted samples were further diluted and re-reacted until the leftover FDVA was more than 20%. The crosstalk between channels for derivatized unlabeled Ala, Ala-^13^C_3_, ^15^N, Ala-2,3,3,3-d_4_ and Ala-3,3,3-d_3_ was evaluated using standards in each experimental run to ensure observed crosstalk did not affect results. All samples were measured at least twice on different days by two independent operators except for islet and acinar tissues due to their low sample amounts.

### Quantification of amino acids

#### LC-MS/MS data analysis

Peak areas integrated by MS Data Viewer software (Bruker) were used for quantification. Calibration curves covered ranges from 0.2-120 pmole for derivatized l-Ala, 0.005-3 pmole for d-Ala, 0.2–200 pmole for d-Ala-^13^C_3_,^15^N, 0.005-2 for l-Ala-3,3,3-d_3_, 0.0025-1 pmole for d-Ala-3,3,3-d_3_, 0.0025-1 pmole for l-Ala-2,3,3,3-d_4_, and 0.1-30 pmole for l-Leu-5,5,5-d_3_ per injection. Peak areas were normalized to the peak area of derivatized l-Ser-^13^C,^15^N present in the same sample by division. Linear regressions were fitted at proper ranges that best cover the detected peak areas in the unknown samples. When necessary, linear regressions were done in two segments for the same analyte to best cover the high and low concentration ranges; for low concentration range calibration curves, the calibration curves were set to intercept at (0,0). All linear regressions achieved R square >0.99 with very several exceptions. After quantification of AAs per injection in unknown samples using peak areas and calibration curves, amounts of un-labeled Ala, isotopically labeled Ala, and l-Leu-5,5,5-d_3_ were calculated for reconstituted samples and blanks. Results of technical replicates were averaged. Analyte recovery after centrifugal filtration step were represented via dividing the amount of l-Leu-5,5,5-d_3_ in unknown samples by the amounts of l-Leu-5,5,5-d_3_ in reference samples, and was used to calculate back the amount of Ala in original sample extraction before filtration. Blank values were then subtracted from corresponding extraction blanks. The concentration of unlabeled and stable isotope labeled Ala were calculated by dividing by different sample metrics (e.g., tissue weight in mg for brain/salivary glands/intestines/pituitary/mouse chow, gut contents weight in mg, μL for plasma, total protein amounts for islets/acinar tissues). The individual processed values can be found in Supplementary Data 1.

#### Data processing

To enable comparisons between two oral administration models and among different sample types, we performed several normalization strategies. To enable comparison between two oral administration scenarios, d-Ala-^13^C_3_,^15^N concentrations in various tissues/gut content in their own metrics (pmole over mg tissues/mg feces/μg proteins) were divided by the plasma d-Ala-^13^C_3_,^15^N level (pmole/μL plasma) (Eq. 1):

1 $${{{{{\rm{Normalized}}}}}}\, {\textsc{d}}-{{{{{\rm{Ala}}}}}}\; {{{{{\rm{isotope}}}}}}\; {{{{{\rm{concentration}}}}}}=\,\frac{{\left[{\textsc{d}}-{{{{{\rm{Ala}}}}}}\; {{{{{\rm{isotope}}}}}}\right]}_{{{{{{{\rm{tissue}}}}}}\; {{{{{\rm{or}}}}}}\; {{{{{\rm{gut}}}}}}\; {{{{{\rm{contents}}}}}}}}}{{\left[{\textsc{d}}-{{{{{\rm{Ala}}}}}}\; {{{{{\rm{isotope}}}}}}\right]}_{{{{{{{\rm{plasma}}}}}}}}{{{{{\rm{of}}}}}}\; {{{{{\rm{the}}}}}}\; {{{{{\rm{same}}}}}}\; {{{{{\rm{animal}}}}}}}$$

To enable comparisons across different sample types, as they have different metrics to represent concentrations, we divided the d-Ala-^13^C_3_,^15^N concentrations to the total unlabeled Ala in each sample to make them unitless. The d-Ala-^13^C_3_,^15^N concentrations were divided by total unlabeled Ala in the same sample, generating ratios of d-Ala-^13^C_3_,^15^N over total unlabeled Ala in the same sample (Eq. 2) as a relative level of d-Ala-^13^C_3_,^15^N:

2 $${\textsc{d}}-{\mbox{Ala isotope ratio}}=\frac{\left[{\textsc{d}}-{\mbox{Ala isotope}}\right]}{\left[{\mbox{total unlabeled Ala}}\right]\, {\mbox{in the same sample}}}$$

Further normalization for Supplementary Fig. 2 was done by dividing such ratio to the plasma d-Ala-^13^C_3_,^15^N ratio to count for biological variations (Eq. 3):

3 $${{{{{\rm{Normalized}}}}}}\, {\textsc{d}}-{{{{{\rm{Ala}}}}}}\; {{{{{\rm{isotope}}}}}}\; {{{{{\rm{ratio}}}}}}=\frac{({{\textsc{d}}-{{{{{\rm{Ala}}}}}}\; {{{{{\rm{isotope}}}}}}\; {{{{{\rm{ratio}}}}}}})_{{{{{{{\rm{tissue}}}}}}\; {{{{{\rm{or}}}}}}\; {{{{{\rm{gut}}}}}}\; {{{{{\rm{concents}}}}}}}}}{({{\textsc{d}}-{{{{{\rm{Ala}}}}}}\; {{{{{\rm{isotope}}}}}}\; {{{{{\rm{ratio}}}}}}})_{{{{{{{\rm{plasma}}}}}}}}{{{{{\rm{of}}}}}}\; {{{{{\rm{the}}}}}}\; {{{{{\rm{same}}}}}}\; {{{{{\rm{animal}}}}}}}$$

### Statistics and reproducibility

Replicates presented in this study refer to animal individuals or tubes of bacteria culture. The data points used in figures and for statistics represent averages of technical replicates from multiple measurements. Non-parametric statistics were implemented for all data obtained from LC-MS measurements. Initially, Welch’s *t* tests were performed due to unequal sample sizes and/or unequal variances. However, due to the small degree of freedom in parametric Welch’s *t* tests resulted from the small sample sizes (*n* = 3-8), non-parametric Mann–Whitney tests were chosen for individual pairwise comparisons. For datasets in Figs. 2,  3c–e,  5b–e, two-tailed Mann–Whitney tests were used. For datasets in Fig. 5f–j, one-tailed Mann–Whitney tests were used because of the sample size (*n* = 3) and the complete separation observed in the dataset (*U* = 0). The non-parametric, paired Friedman’s one-way ANOVA followed by Dunn’s multiple comparison were used to determine if tissue types significantly contribute to d-Ala-^13^C_3_,^15^N level and to investigate the differences among tissue types (Fig. 3f, g). For peptide profiling data (Fig. 4), uncorrected multiple *t* tests with more assumptions were selected to maximize the number of discoveries for future studies due to the semi-quantitative nature of MALDI-TOF MS, the small sample size (*n* = 4), and the exploratory nature of this experiment. The significance level (α) for all statistics is set to 0.05 when describing statistical significance. All statistics were done in GraphPad Prism 9.2.0.

### Reporting summary

Further information on research design is available in the Nature Portfolio Reporting Summary linked to this article.

### Supplementary information

SI file

Description of Supplementary Materials

Supplementary Data 1

Reporting Summary

## Data Availability

Source data for all graphs and charts can be found in the Supplementary Data file. The processed data supporting the conclusions are presented in the main text and Supplementary Information. The original data are available and will be shared upon request by contacting the corresponding author or the lead author (qiutian8@msu.edu).
