# Supplementary material for: Biodistribution and racemization of gut-absorbed l/d-alanine in germ-free mice
Source: Commun Biol. 2023 Aug 16;6:851. doi: 10.1038/s42003-023-05209-y (PMC10432453; doi:10.1038/s42003-023-05209-y)
Supplement: Supplementary file 1 — SI file [file 42003_2023_5209_MOESM1_ESM.docx]

**Supplementary Information**

**Biodistribution and racemization of gut-absorbed l/d-alanine in germ-free mice**

Tian (Autumn) Qiu^1,2,#^, Cindy J. Lee^1^, Chen Huang^3^, Dong-Kyu Lee^1,*^, Stanislav S. Rubakhin^1,2,3^, Elena V. Romanova^1,2,3^, Jonathan V. Sweedler^1,2,3^

^1^Department of Chemistry, University of Illinois Urbana-Champaign, Urbana, IL 61801

^2^Beckman Institute, University of Illinois Urbana-Champaign, Urbana, IL 61801

^3^Neuroscience Program, University of Illinois Urbana-Champaign, Urbana, IL 61801

^*^ Current address: College of Pharmacy, Chung-Ang University, Seoul, 06974, Republic of Korea

^#^ Current address: Department of Chemistry, Michigan State University, East Lansing, MI 48824

E-mail address of the corresponding author: [jsweedle@illinois.edu](mailto:jsweedle@illinois.edu)

**Table of Contents**

**Supplementary Figure 1**. d-Ala-^13^C_3_,^15^N ratios to unlabeled Ala in different sample types, plotted by individual animals numbered as Rep 1-4. Connected lines indicate the same sample type across animals.

**Supplementary Table 1**. Pearson correlation of d-Ala-^13^C_3_,^15^N ratios in plasma vs. other types of samples.

**Supplementary Figure 2**. Relative d-Ala-^13^C_3_,^15^N level (Equation 3) in various sample types.

**Supplementary Figure 3**. MRM spectra of d-Ala-3,3,3-D_3_ for salivary glands.

**Supplementary Figure 4**. Relative d-Ala-3,3,3-d_3_ levels (Equation 2) in various sample types in l-Ala-2,3,3,3-d_4_ gavage experiments to conventional mice.

**Supplementary Table 2**. A summary of d-Ala concentration reported in regular and germ-free rodents.

**Supplementary Figure 5**. Phylogram from multisequence alignment of mouse serine racemase and several selective amino acid racemases from prokaryotes, yeast and invertebrates.

**Supplementary Table 3**. Enantiomeric impurities (%) in standards used for oral administration.

**Supplementary Figure 6.** Illustration and example separation of derivatized alanine.

**Supplementary Table 4**. Source parameters.

**Supplementary Table 5**. MRM transitions and typical retention time of derivatized amino acids.

**Supplementary Figure 1**. d-Ala-^13^C_3_,^15^N ratios (Equation 1) to unlabeled Ala in different sample types, plotted by individual animals numbered as Rep 1-4. Connected lines indicate the same sample type across animals. Due to limited space, not all sample types are marked.

**Supplementary Table 1**. Pearson correlation of d-Ala-^13^C_3_,^15^N ratios in plasma vs. other types of samples from the same animal individuals.

| **Gavage** | | Plasma vs. Pituitary | | Plasma vs. Brain | | Plasma vs. Small int | | Plasma vs. SI Content | | Plasma vs. Colon | | Plasma vs. Colon content | | Plasma vs. Islets | | Plasma vs. Acinar | |
| --- | --- | --- | --- | --- | --- | --- | --- | --- | --- | --- | --- | --- | --- | --- | --- | --- | --- |
| r | | 0.9756 | | 0.9520 | | 0.9929 | | 0.9844 | | 0.9810 | | 0.3361 | | -0.4048 | | 0.9975 | |
| P (two-tailed) | | 0.0244 | | 0.0480 | | 0.0071 | | 0.0156 | | 0.0190 | | 0.6639 | | 0.5952 | | 0.0025 | |
| P value | | * | | * | | ** | | * | | * | | ns | | ns | | ** | |
| Number of XY Pairs | | 4 | | 4 | | 4 | | 4 | | 4 | | 4 | | 4 | | 4 | |
|  | |  | |  | |  | |  | |  | |  | |  | |  | |
| **Feeding** | Plasma vs. Pituitary | | Plasma vs. Brain | | Plasma vs. Salivary gland | | Plasma vs. Small int | | Plasma vs. SI Content | | Plasma vs. Colon | | Plasma vs. Colon content | | Plasma vs. Islets | | Plasma vs. Acinar |
| r | 0.9813 | | -0.1407 | | 0.9446 | | 0.9986 | | 0.9979 | | 0.9908 | | 0.9758 | | -0.5419 | | 0.4425 |
| P (two-tailed) | 0.0187 | | 0.8593 | | 0.0554 | | 0.0014 | | 0.0021 | | 0.0092 | | 0.0242 | | 0.4581 | | 0.5575 |
| P value | * | | ns | | ns | | ** | | ** | | ** | | * | | ns | | ns |
| Number of XY Pairs | 4 | | 4 | | 4 | | 4 | | 4 | | 4 | | 4 | | 4 | | 4 |

**Supplementary Figure 2**. Relative d-Ala-^13^C_3_,^15^N level (**Equation 3**) in various sample types in (c) gavage and (d) feeding experiments. Asterisks with brackets indicate statistically significant difference (p<0.05) between sample types from the post hoc Dunn’s multiple comparison after Kruskal-Wallis ANOVA (** p<0.01, * p<0.05). Each data point represents one sample from one individual animal (n=4, except for n=2 for salivary gland in gavage and n=3 for acinar in gavage). Floating bars indicate minimum and maximum, and the middle lines indicate median. Salivary gland results in gavage experiment are not shown in (b)(c) and not included in statistics due to small sample sizes (n=2). N/S: not shown. SI – small intestine.


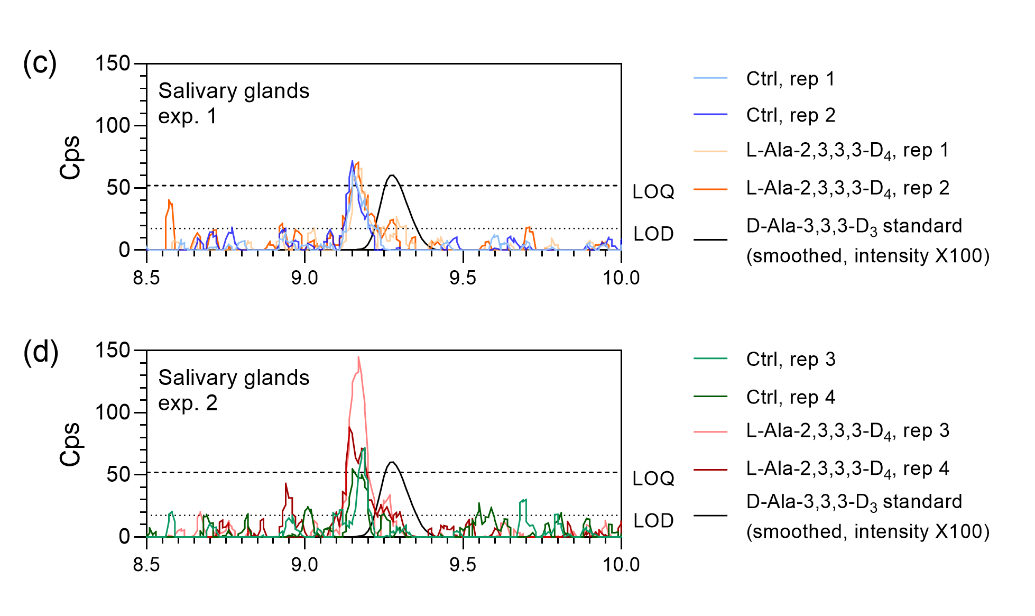


**Supplementary Figure 3**. MRM spectra of d-Ala-3,3,3-d_3_ for salivary glands. The top extracted ion chromatogram showed the samples from the first dosing experiment (replicate 1 and 2 in each treatment group) and the bottom one showed the second dosing experiment (replicate 3 and 4). Limit of detection (LOD) and limit of quantification (LOQ) were quantified by 3 or 10 times the standard deviation of baseline intensity from 8.5-9 min and 9.5-10 min.

**Supplementary Figure 4**. Relative d-Ala-3,3,3-d_3_ levels (**Equation 2**) in various sample types in l-Ala-2,3,3,3-d_4_ gavage experiments to conventional mice. Asterisks with brackets indicate statistically significant difference (p<0.05) between sample types from the post hoc Dunn’s multiple comparison after Friedman’s paired ANOVA (* p<0.05). Each data point represents one sample from one individual animal. Floating bars indicate minimum and maximum, and the middle lines indicate median. SI – small intestine.

**Supplementary Table 2**. A summary of d-Ala concentration in conventional and germ-free rodents observed here compared to values reported in previous studies. NA: not measured or included in the study. Unknown: measured but the value was not reported. Not comparable: reported in different units compared to this study. Numbers with “~” indicate an estimated value from graphs.

|  | Plasma (pmole/μL plasma) | | | | | | | Pancreas (pmole/mg tissue) | | | Feces (pmole/mg feces) | |
| --- | --- | --- | --- | --- | --- | --- | --- | --- | --- | --- | --- | --- |
|  | Karakawa, 2013 (1) | Bruckner, 2001 (2) | Weatherly, 2017 (blood) (3) | Hashimoto, 1993 (serum) (4) | Nagata, 1994 (serum) (5) | Miyoshi, 2009 (serum) (6) | This work | Karakawa, 2013 (1) | Miyoshi, 2009 (6) | This work (acinar) | Bruckner, 2001 (2) | This work (colon content) |
| **Conventional** | Male ICR mice | Male white mice | NIH Swiss mice | Male mice | Male mice | Male mice | Male C57BL/6 mice | Male ICR mice | Male mice | Male C57BL/6 mice | Male white mice | Male C57BL/6 mice |
| D-Ala | ~8 | Unknown | Not comparable | 6 | 2.7 | ~10 | 1.903 | ~65 | ~125 | 0.099 | Unknown | 528 |
| D% | ~1.4% | 1.00% | 0.008-0.2% | 1.50% | 0.74% | ~2.8% | 0.37% | ~1.2% | Unknown | 0.48% | 5.90% | 26.20% |
| **Germ-free** | Male ICR mice | Male Wistar rats | -- | -- | -- | -- | Male C57BL/6 mice | Male ICR mice | -- | Male C57BL/6 mice | Male RII/Tif rat | Male C57BL/6 mice |
| D-Ala | ~0.4 | 1.1 | NA | NA | NA | NA | 1.271 | ~4 | NA | 0.024 | 71 (Rats) | 10.67 |
| D% | Unknown | 0.4% | NA | NA | NA | NA | 0.12% | Unknown | NA | 0.55% | 1% (Rats) | 0.28% |

|  | Pituitary (pmole/mg tissue) | | | | Brain (pmole/mg tissue) | | | | Salivary glands (pmole/mg tissue) | |
| --- | --- | --- | --- | --- | --- | --- | --- | --- | --- | --- |
|  | Karakawa, 2013 (1) | Morikawa, 2001 (7) | Miyoshi, 2009 (6) | This work | Morikawa, 2001 (different regions) (7) | Weatherly, 2017 (perfused, hippocampus/cortex) (3) | Miyoshi, 2009 (6) | This work | Yoshikawa, 2022 (8) | This work |
| **Conventional** | Male ICR mice | Male mice | Male mice | Male C57BL/6 mice | Male mice | NIH Swiss mice | Male mice | Male C57BL/6 mice | Male Wistar rats | Male C57BL/6 mice |
| D-Ala | ~10 | 29.1 | ~15 | 2.881 | 12.4/11.4/90/10.9/4.6/36.5 | 382/281 | n.d.-10.9/n.d.-12.4 | 1.92 | 11.6/14.1/13.0 | 4.37 |
| D% | ~0.36% | 1.90% | ~0.31% | 0.23% | 0.7/0.6/0.6/1.2/0.5/2.4% | 13.0/9.8% | Unknown | 0.12% | 0.3/0.2/0.2% | 0.11% |
| **Germ-free** | Male ICR mice | -- | -- | Male C57BL/6 mice | -- | -- | -- | Male C57BL/6 mice | -- | Male C57BL/6 mice |
| D-Ala | ~4.5 | NA | NA | 1.932 | NA | NA | NA | 1.46 | NA | 3.63 |
| D% | Unknown | NA | NA | 0.11% | NA | NA | NA | 0.11% | NA | 0.07% |

**
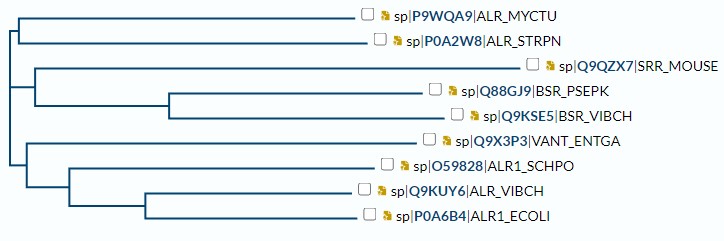
**

**Supplementary Figure 5**. Phylogram from multisequence alignment of mouse serine racemase and several selective amino acid racemases from prokaryotes, yeast and invertebrates using the UniProt Align tool.

**Supplementary Table 3**. Enantiomeric impurities (%) in standards used for oral administration. The levels of impurity were estimated based on retention time, peak area, calibration curve of d/l-Ala-3,3,3-d_3_, and estimated crosstalk between MRM channels.

| **Standards for feeding** | **Condition** | **Impurity** | **Impurity%** |
| --- | --- | --- | --- |
| d-Ala-^13^C_3_,^15^N | As-made | l-Ala-^13^C_3_,^15^N | 1.47% |
|  | Autoclaved once | l-Ala-^13^C_3_,^15^N | 1.56% |
|  | Autoclaved twice | l-Ala-^13^C_3_,^15^N | 1.17% |
|  | Leftover after 2-wk | l-Ala-^13^C_3_,^15^N | 1.76% |
| l-Ala-2,3,3,3-d_4_ | As-made | d-Ala-2,3,3,3-d_4_ | 0.27% |
|  | Autoclaved once | d-Ala-2,3,3,3-d_4_ | 0.17% |
|  | Autoclaved twice | d-Ala-2,3,3,3-d_4_ | 0.29% |
|  | Leftover after 2-wk | d-Ala-2,3,3,3-d_4_ | 0.28% |
|  | As-made | d-Ala-3,3,3-d_3_ | 0.02% |
|  | Autoclaved once | d-Ala-3,3,3-d_3_ | 0.12% |
|  | Autoclaved twice | d-Ala-3,3,3-d_3_ | 0.12% |
|  | Leftover after 2-wk | d-Ala-3,3,3-d_3_ | 0.11% |

**
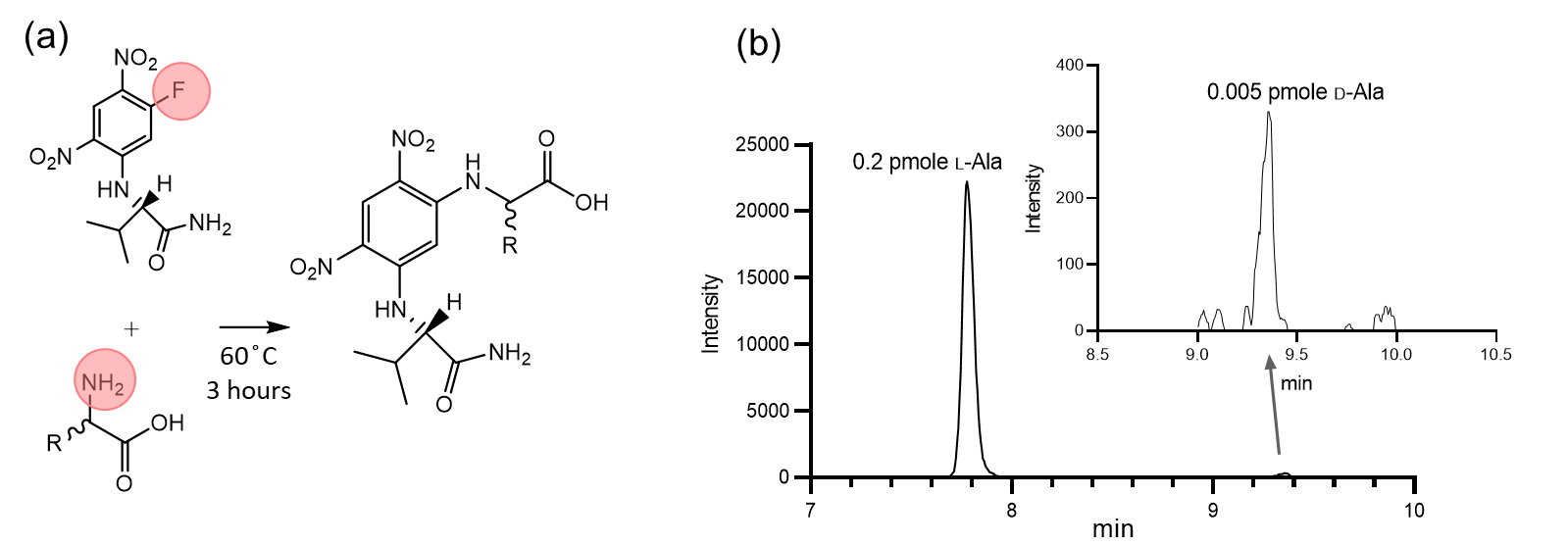
**

**Supplementary Figure 6.** Illustration and example separation of derivatized alanine. (a) An illustration of *N*_α_-(2,4-dinitro-5-fluorophenyl)-l-valinamide (FDVA) reacting with amino acids. (b) Demonstration of separation of derivatized l- and d-Ala.

**Supplementary Table 4**. Source parameters of the triple quadrupole mass spectrometer for amino acid analysis.

| Source | HESI |
| --- | --- |
| **Spray Voltage (-)** | 3500 V |
| **Cone Temperature** | 250 °C |
| **Cone Gas Flow** | 20 |
| **Heated Probe Temperature** | 400 °C |
| **Heated Probe Gas Flow** | 45 |
| **Nebulizer Gas Flow** | 50 |
| **Exhaust Gas** | OFF |

**Supplementary Table 5**. MRM transitions and typical retention time of derivatized amino acids and the derivatization reagent (FDVA) at negative mode detection. The retention time (RT) range was estimated from raw spectra.

|  |  |  |  | RT, min |  |
| --- | --- | --- | --- | --- | --- |
|  | **Precursor, *m/z* (Q1 resolution)** | **Product, *m/z* (Q3 resolution)** | **Collision energy, eV** | **FDVA-l-AA** | **FDVA-d-AA** |
| **FDVA** | 299.2 (0.7) | 195.0 (0.7) | 12.0 | 10.15-10.17 |  |
|  |  | 165.0 (0.7) | 19.0 |  |  |
|  |  | 220.0 (0.7) | 14.0 |  |  |
| **FDVA-Ala** | 368.3 (0.7) | 288.7 (2.0) | 11.0 | 7.78-7.80 | 9.35-9.37 |
|  |  | 173.8 (2.0) | 33.0 |  |  |
| **FDVA-d-Ala-^13^C_3_,^15^N** | 372.3 (0.7) | 292.2 (0.7) | 11.0 |  | 9.35-9.37 |
|  |  | 176.1 (0.7) | 31.0 |  |  |
|  |  | 209.1 (0.7) | 12.0 |  |  |
| **FDVA-l-Ala-2,3,3,3-d_4_** | 372.3 (0.6) | 291.2 (0.6) | 10.0 | 7.77-7.80 |  |
|  |  | 175.2 (0.6) | 31.0 |  |  |
|  |  | 191.1 (0.6) | 22.0 |  |  |
| **FDVA-d-Ala-3,3,3-d_3_** | 371.3 (0.6) | 291.2 (0.6) | 10.0 |  | 9.33-9.36 |
|  |  | 209.1 (0.6) | 12.0 |  |  |
|  |  | 191.1 (0.6) | 22.0 |  |  |
| **FDVA-l-Ser-^13^C,^15^N** | 386.3 (0.6) | 163.1 (0.6) | 24.0 | 7.22-7.24 |  |
|  |  | 193.1 (0.6) | 14.0 |  |  |
|  |  | 293.1 (0.6) | 8.0 |  |  |
| **FDVA-L-Leu-5,5,5-d_3_** | 413.4 (0.7) | 351.2 (0.7) | 7.0 | 9.28-9.30 |  |
|  |  | 174.0 (0.7) | 32.0 |  |  |
|  |  | 291.0 (0.7) | 9.0 |  |  |

**Supplementary References:**

1. Karakawa, S., Miyoshi, Y., Konno, R., Koyanagi, S., Mita, M., Ohdo, S., and Hamase, K. (2013) Two-dimensional high-performance liquid chromatographic determination of day–night variation of d-alanine in mammals and factors controlling the circadian changes. *Anal Bioanal Chem*. **405**, 8083–8091

2. Brückner, H., and Schieber, A. (2001) Ascertainment of d-amino acids in germ-free, gnotobiotic and normal laboratory rats. *Biomed Chromatogr*. **15**, 257–262

3. Weatherly, C. A., Du, S., Parpia, C., Santos, P. T., Hartman, A. L., and Armstrong, D. W. (2017) d-Amino Acid Levels in Perfused Mouse Brain Tissue and Blood: A Comparative Study. *ACS Chem. Neurosci.* **8**, 1251–1261

4. Hashimoto, A., Nishikawa, T., Konno, R., Niwa, A., Yasumura, Y., Oka, T., and Takahashi, K. (1993) Free d-serine, d-aspartate and d-alanine in central nervous system and serum in mutant mice lacking d-amino acid oxidase. *Neurosci Letters*. **152**, 33–36

5. Nagata, Y., Konno, R., and Niwa, A. (1994) Amino acid levels in d-alanine-administered mutant mice lacking d-amino acid oxidase. *Metabolism*. **43**, 1153–1157

6. Miyoshi, Y., Hamase, K., Tojo, Y., Mita, M., Konno, R., and Zaitsu, K. (2009) Determination of d-serine and d-alanine in the tissues and physiological fluids of mice with various d-amino-acid oxidase activities using two-dimensional high-performance liquid chromatography with fluorescence detection. *J Chromatogr B*. **877**, 2506–2512

7. Morikawa, A., Hamase, K., Inoue, T., Konno, R., Niwa, A., and Zaitsu, K. (2001) Determination of free d-aspartic acid, d-serine and d-alanine in the brain of mutant mice lacking d-amino-acid oxidase activity. *J Chromatogr B Biomed Appl*. **757**, 119–125

8. Yoshikawa, M., Kan, T., Shirose, K., Watanabe, M., Matsuda, M., Ito, K., and Kawaguchi, M. (2022) Free d-Amino Acids in Salivary Gland in Rat. *Biology*. **11**, 390
