# Supplementary material for: Biodistribution and racemization of gut-absorbed l/d-alanine in germ-free mice
Source: Commun Biol. 2023 Aug 16;6:851. doi: 10.1038/s42003-023-05209-y (PMC10432453; doi:10.1038/s42003-023-05209-y)
Supplement: Supplementary file 2 — Description of Supplementary Materials [file 42003_2023_5209_MOESM2_ESM.docx]

**Description of Additional Supplementary Files**

**File name:** Supplementary Data 1

**Description:** The source data in this paper. Including: “Amino acid analysis” for raw data of amino acid quantification, “Amino acid processed” for processed, normalized data, “Source data for Figures” for source data of amino acid quantification behind the graphs, and “Identified peptides” for a list of identified peptides using MALDI-TOF.
