# Supplementary material for: Biodistribution and racemization of gut-absorbed l/d-alanine in germ-free mice
Source: Commun Biol. 2023 Aug 16;6:851. doi: 10.1038/s42003-023-05209-y (PMC10432453; doi:10.1038/s42003-023-05209-y)
Supplement: Supplementary file 4 — Reporting Summary [file 42003_2023_5209_MOESM4_ESM.pdf]

nature portfolio

Corresponding author(s): Jonathan V. Sweedler

Last updated by author(s): Jul 22, 2023

Reporting Summary

Nature Portfolio wishes to improve the reproducibility of the work that we publish. This form provides structure for consistency and transparency in reporting. For further information on Nature Portfolio policies, see our Editorial Policies and the Editorial Policy Checklist.

Statistics

For all statistical analyses, confirm that the following items are present in the figure legend, table legend, main text, or Methods section.

n/a	Confirmed	
☐	☒	The exact sample size (*n*) for each experimental group/condition, given as a discrete number and unit of measurement
☐	☒	A statement on whether measurements were taken from distinct samples or whether the same sample was measured repeatedly
☐	☒	The statistical test(s) used AND whether they are one- or two-sided<br>*Only common tests should be described solely by name; describe more complex techniques in the Methods section.*
☒	☐	A description of all covariates tested
☐	☒	A description of any assumptions or corrections, such as tests of normality and adjustment for multiple comparisons
☐	☒	A full description of the statistical parameters including central tendency (e.g. means) or other basic estimates (e.g. regression coefficient) AND variation (e.g. standard deviation) or associated estimates of uncertainty (e.g. confidence intervals)
☐	☒	For null hypothesis testing, the test statistic (e.g. *F*, *t*, *r*) with confidence intervals, effect sizes, degrees of freedom and *P* value noted<br>*Give P values as exact values whenever suitable.*
☒	☐	For Bayesian analysis, information on the choice of priors and Markov chain Monte Carlo settings
☒	☐	For hierarchical and complex designs, identification of the appropriate level for tests and full reporting of outcomes
☒	☐	Estimates of effect sizes (e.g. Cohen's *d*, Pearson's *r*), indicating how they were calculated

Our web collection on statistics for biologists contains articles on many of the points above.

Software and code

Policy information about availability of computer code

Data collection	Bruker MSWS was used to collect LC-MS data and Bruker flexControl was used to collect MALDI-MS data.
Data analysis	GraphPad Prism 9.4.0 (673) was used to analyze the data.

For manuscripts utilizing custom algorithms or software that are central to the research but not yet described in published literature, software must be made available to editors and reviewers. We strongly encourage code deposition in a community repository (e.g. GitHub). See the Nature Portfolio guidelines for submitting code & software for further information.

Data

Policy information about availability of data

All manuscripts must include a data availability statement. This statement should provide the following information, where applicable:

- Accession codes, unique identifiers, or web links for publicly available datasets
- A description of any restrictions on data availability
- For clinical datasets or third party data, please ensure that the statement adheres to our policy

The authors declare that the processed data supporting the findings of this study are available within the article (and its supplementary information files). The original datasets generated during this study are available from the corresponding author on reasonable request.

Human research participants

Policy information about studies involving human research participants and Sex and Gender in Research.

Reporting on sex and gender	N/A
Population characteristics	N/A
Recruitment	N/A
Ethics oversight	N/A

Note that full information on the approval of the study protocol must also be provided in the manuscript.

Field-specific reporting

Please select the one below that is the best fit for your research. If you are not sure, read the appropriate sections before making your selection.

☒ Life sciences ☐ Behavioural & social sciences ☐ Ecological, evolutionary & environmental sciences

For a reference copy of the document with all sections, see nature.com/documents/nr-reporting-summary-flat.pdf

Life sciences study design

All studies must disclose on these points even when the disclosure is negative.

Sample size	Due to the high price of germ-free mice and stable isotopes we used, we chose to use the minimal acceptable sample size (n=3 or 4 for each group) and investigate whether effects can be seen with a small sample size.
Data exclusions	No data exclusion except for several data points for islets and acinar tissues. In four control islet samples (I51-52, I31-32), no L-Ala was detected, and thus D-Ala percentage cannot be calculated; these data points were excluded. In one acinar sample (A23), partial sample was lost during sample preparation, so only percentage was kept but the absolute values were invalidated. In another acinar sample (A31), the endogenous L-Ala was too close to the background level that makes all calculated ratios outliers (determined by Grubb's test); the data points associated with this sample were excluded.
Replication	All experiments were performed in different animals or cohort of animals. All samples, except for islet and acinar tissues (due to low sample amounts), were measured at least twice by two different operators.
Randomization	In each experiment, we randomly choose the mice or the cage of mice to perform control or treatment. The sample collection, preparation, and measurement were not possible to be randomized since it is necessary to process control and treated samples separately to avoid cross-contamination.
Blinding	Blinding during experiment was not possible because we had to process control and treated animals/samples separately to avoid cross-contamination. However, the raw data collection and the analysis were done by two different operators to avoid biases during data analysis.

Reporting for specific materials, systems and methods

We require information from authors about some types of materials, experimental systems and methods used in many studies. Here, indicate whether each material, system or method listed is relevant to your study. If you are not sure if a list item applies to your research, read the appropriate section before selecting a response.

Materials & experimental systems

n/a	Involved in the study	
☒	☐	Antibodies
☒	☐	Eukaryotic cell lines
☒	☐	Palaeontology and archaeology
☐	☒	Animals and other organisms
☒	☐	Clinical data
☒	☐	Dual use research of concern

Methods

n/a	Involved in the study	
☒	☐	ChIP-seq
☒	☐	Flow cytometry
☒	☐	MRI-based neuroimaging

Animals and other research organisms

Policy information about studies involving animals; ARRIVE guidelines recommended for reporting animal research, and Sex and Gender in Research

Laboratory animals	Germ-free C57BL/6 mice (9-11 weeks old), conventional C57BL/6 mice (8 weeks old)
Wild animals	N/A
Reporting on sex	This study was done on only male mice. Sex was considered during study design. As this study involves peptide hormone analysis, males and females needs to be separately investigated due to their different hormone profiles and cycles. We decided to perform the study only using male mice, and plan a follow up with further investigation on female mice.
Field-collected samples	N/A
Ethics oversight	Animal experiments were performed following the animal use protocol #19251 approved by the Institutional Animal Care and Use Committee (IACUC) at the University of Illinois Urbana-Champaign (UIUC).

Note that full information on the approval of the study protocol must also be provided in the manuscript.
